# Time for a new global roadmap for supporting evidence into action

**DOI:** 10.1371/journal.pgph.0000677

**Published:** 2022-06-23

**Authors:** Tanja Kuchenmüller, John Lavis, Mehrnaz Kheirandish, Ludovic Reveiz, Marge Reinap, Joseph Okeibunor, Siswanto Siswanto, Arash Rashidian, Samuel Sieber, Kaelan Moat, Cristian Mansilla, Fadi El-Jardali, Matthias Helble, John Reeder, Evelina Chapman, Jorge Otávio Maia Barreto, Ahmed Mandil, Soumya Swaminathan

**Affiliations:** 1 Science Division, Department of Research for Health, World Health Organization, Geneva, Switzerland; 2 McMaster Health Forum/WHO Collaborating Centre for Evidence-Informed Policy, and Department of Health Evidence and Impact, McMaster University, Hamilton, Canada; 3 Department of Science, Information and Dissemination, WHO Regional Office for the Eastern Mediterranean, Cairo, Egypt; 4 Department of Evidence and Intelligence for Action in Health and Incident Management System for COVID-19, WHO Regional Office for the Americas/Pan American Health Organization, Washington, DC, United States of America; 5 Division of Country Health Policies and System, WHO Regional Office for Europe, Copenhagen, Denmark; 6 Research Development and Innovation, Assistant Regional Director’s Cluster, WHO Regional Office for Africa, Brazzaville, Republic of Congo; 7 Healthier Population and Non-Communicable Disease Department, WHO Regional Office for South-East Asia, New Delhi, India; 8 Knowledge to Policy (K2P) Center/WHO Collaborating Centre for Evidence-Informed Policymaking and Practice, and Department of Health Management and Policy, American University of Beirut, Beirut, Lebanon; 9 Special Programme for Research and Training in Tropical Diseases, World Health Organization, Geneva, Switzerland; 10 Oswaldo Cruz Foundation, Brasilia, Brazil; 11 Office of Chief Scientist, World Health Organization, Geneva, Switzerland; PLOS: Public Library of Science, UNITED STATES

The COVID-19 pandemic has placed the use of evidence for policy-making high up on the international agenda. To fight the pandemic, Governments around the world have publicly stressed the need to draw on evidence by engaging scientific advisors and advisory bodies [1]. Furthermore, the increased demand for evidence has led to a global push for innovative solutions such as the scaling-up of living evidence syntheses [2]. At the same time, COVID-19 revealed fatal structural and systemic weaknesses in the production and use of evidence–flaws which have cost lives [3]. In many cases, institutional mechanisms and capacities to systematically mobilize and contextualize the best available evidence for rapid decision-making were missing [4]. As a consequence, policy-makers, practitioners and citizens alike were confronted with a deluge of competing claims and misinformation, severely limiting suitable decision-making and taking action [5]. The related surge of vaccine hesitancy has disproportionally impacted ethnic minorities and deprived communities, with the lowest vaccine uptake, worryingly, to be seen among the most vulnerable people—the older, the more clinically vulnerable, and those living in the most deprived areas—worsening pre-existing disparities in vaccine use, health inequalities and socio-economic marginalization [6, 7].

To assess different institutional responses in terms of the evidence-policy-society nexus and to learn lessons on how to build equity-centred, agile and responsive evidence-informed decision-making mechanisms, WHO convened its first Global Evidence-to-Policy Summit [8] in late 2021. The Summit, organized by the newly created Evidence to Policy Unit at WHO headquarters in collaboration with the corresponding teams in WHO regional offices, brought together more than 2,500 policy-makers, knowledge brokers, health actors, civil society representatives and researchers from around the world.

At the Summit, WHO Director-General, Tedros Adhanom Ghebreyesus [9], was joined by keynote speakers such as Angela Merkel [10] (former Chancellor of Germany), Michelle Bachelet [11] (United Nations High Commissioner for Human Rights), and Ban Ki-moon (former Secretary-General of the United Nations); as well as health ministers and representatives of renowned national and international academic institutions calling for the urgent need to redesign and strengthen the global evidence-policy-society ecosystem. Drawing on experiences and insights gained during the pandemic, the Summit formulated clear demands to increase the use of evidence, appealing to the international community to use evidence to promote human rights, reverse existing inequalities exacerbated by COVID-19 and win back developmental progress towards the Sustainable Development Goals (SDGs). Promising strategies to improve evidence-informed decision-making and create a more sustainable, equitable and resilient world post-COVID-19 include:

- strengthening collaboration, information sharing and networking at country, regional and global levels to reduce duplication of efforts in evidence generation and widely increase access to the benefits of research evidence;
- moving away from siloed approaches and establishing integrated systems to leverage multisectoral and multidisciplinary approaches, beyond research;
- creating and/or enhancing national structures and evidence advisory bodies that swiftly respond to decision-makers’ requirements for evidence;
- creating and/or scaling-up innovative mechanisms that allow decision-makers to keep abreast of large amounts of rapidly changing evidence, such as living evidence syntheses; and
- raising public trust in evidence to improve pandemic response efforts and accelerate the achievement of the 2030 Agenda and SDGs.

WHO’s Evidence-Informed Policy Network (EVIPNet) [12], a long-standing global initiative promoting the systematic use of the best global and local evidence in policy-making in more than 50 countries around the world, was in the Summit’s spotlight. Five regional sessions organized by the WHO Regional Offices in Africa, the Americas, the Eastern Mediterranean (with its Regional Network of Institutions for Evidence and Data to Policy (NEDtP), Europe and South-East Asia and opened by the respective WHO (Assistant) Regional Directors, featured important insights into locally contextualized evidence-to-policy/society approaches. These included a series of case studies on how countries responded to regional and local challenges of the pandemic. The Evidence Center at the Brazilian Ministry of Health, for instance, produced more than 80 rapid syntheses and 20 plain language summaries in response to COVID-19, applying agile, innovative knowledge translation approaches to target decision-makers’ needs.

The Summit culminated in the launch of the EVIPNet Call for Action [13], developed through iterative, participatory processes with EVIPNet members, and the announcement of a new Coalition of Partners for sustainable evidence-to-policy/society systems. The Call, aligned with the Cochrane Convenes [14] and the Global Commission on Evidence to Address Societal Challenges [4], urges governments, intergovernmental organizations such as United Nations agencies, international financial institutions and other key stakeholders to join forces and commit to 16 concrete steps towards better, equity-centered evidence-informed decision-making. In four strategic sections, the Call focuses on:

1. institutionalizing structures, such as the EVIPNet knowledge translation platforms, which serve as institutional knowledge intermediaries that are agile and can rapidly respond to societal and decision-makers’ needs, while drawing on different forms of contextualized, actionable evidence;
2. developing and adhering to high-quality norms and standards that promote rigorous, transparent and systematic evidence-to-policy-society mechanisms and rely on equity-oriented, inclusive and multisectoral participation of all stakeholders;
3. strengthening national and international capacity, including sustainable funding, to empower countries and international actors in evidence-to-policy-society processes, while enhancing legitimacy and building trusted relationships across the evidence ecosystem between researchers, policy-makers and the society; and
4. striving to ensure that global public goods, such as relevant, timely and high-quality global evidence syntheses and locally adaptable guidelines, are available and easily accessible, especially in emergency situations.

Senior leaders of key stakeholder and partner institutions–including the Asian Development Bank, Cochrane, the Evidence Commission, UNICEF Innocenti, among others–expressed strong support for the Call for Action and committed to joining the new Coalition of Partners to foster sustainable evidence-to-policy-society systems.

Mobilizing evidence for better decision-making is certainly not a novel field. Yet, the COVID-19 pandemic has put the spotlight on the importance of evidence. It has ‘stress-tested’ [3] the evidence ecosystem, revealing its systemic weaknesses and failures in both domestic evidence infrastructures and the global evidence architecture. To leverage the power of science and evidence in support of an equitable COVID-19 recovery process and increase country resilience against the current and future crises, collaborative, integrative evidence-policy-society efforts need to take a leap towards more mature institutionalization. Whereas the EVIPNet Call for Action provides the technical and strategic roadmap, the most pressing endeavor now is to step-up efforts, garner political commitment and mobilize investments to put in place the national, regional and global implementation plans of the Call. Governments, intergovernmental organizations and other key stakeholders, such as bi-/multilateral donors and foundations, will need to amalgamate and consolidate efforts and resources, including sustainable financing, along with governments’ commitment for active implementation. Only then can the use of evidence become routinized and make a meaningful difference to people’s lives, in and beyond health crises.
